# Anti-tumor and Anti-angiogenic Ergosterols from *Ganoderma lucidum*

**DOI:** 10.3389/fchem.2017.00085

**Published:** 2017-10-30

**Authors:** Shaodan Chen, Tianqiao Yong, Yifang Zhang, Jiyan Su, Chunwei Jiao, Yizhen Xie

**Affiliations:** ^1^Guangdong Institute of Microbiology, State Key Laboratory of Applied Microbiology Southern China, Guangdong Provincial Key Laboratory of Microbial Culture Collection and Application, Guangdong Open Laboratory of Applied Microbiology, Guangzhou, China; ^2^Yuewei Edible Fungi Technology Co. Ltd., Guangzhou, China

**Keywords:** *Ganoderma lucidum*, ergosterols, anti-tumor, anti-angiogenic, QSAR

## Abstract

This study was carried out to isolate chemical constituents from the lipid enriched fraction of *Ganoderma lucidum* extract and to evaluate their anti-proliferative effect on tumor cells and human umbilical vein endothelial cells (HUVECs). Ergosterol derivatives (**1**–**14**) were isolated and purified from the lipid enriched fraction of *G. lucidum*. Their chemical structures were established by spectroscopic analyses or by comparison of mass and NMR spectral data with those reported previously. Amongst, compound **1** was purified and identified as a new one. All the compounds were evaluated for their anti-proliferative effect on human tumor cells and HUVECs *in vitro*. Compounds **9–13** displayed inhibitory activity against two types of human tumor cells and HUVECs, which indicated that these four compounds had both anti-tumor and anti-angiogenesis activities. Compound **2** had significant selective inhibition against two tumor cell lines, while **3** exhibited selective inhibition against HUVECs. The structure–activity relationships for inhibiting human HepG2 cells were revealed by 3D-QASR. Ergosterol content in different parts of the raw material and products of *G. lucidum* was quantified. This study provides a basis for further development and utilization of ergosterol derivatives as natural nutraceuticals and functional food ingredients, or as source of new potential antitumor or anti-angiogenesis chemotherapy agent.

## Introduction

Tumor, characterized by abnormal cell proliferation and metastasis, is one of the most attracted chronic diseases and remains the leading cause of death worldwide. Tumor development is accompanied by tumor angiogenesis. Angiogenesis plays a key role in tumor formation, growth, invasion, and metastasis. Inhibiting the angiogenesis of tumors can not only cut off the supply of nutrients such as, oxygen and nutrients needed for tumor growth, but also cut the way of tumor cell metastasis (Folkman, 1971; Papetti and Herman, 2002; McDougall et al., 2006; Berz and Wanebo, 2011). Therefore, inhibitors of tumor angiogenesis are considered to be an effective strategy for the treatment of cancer.

Back-to-nature has become a chasing idea in recent decades. Edible or medicinal fungi have attracted more and more attention due to their extensive pharmacological effect for chronic diseases (Loria-Kohen et al., 2014), especially for preventing or complementary treating cancer. As one of the most famous medicinal fungi, *Ganoderma lucidum* (Ganodermataceae), firstly recorded in *Shen Nong's Herbal Classic*, was believed by the ancient people to promote longevity and even revive the dead with beautiful legends. The mystery of *G. lucidum* aroused widespread interest of researchers in modern times. *G. lucidum* was demonstrated to possess activities including anti-cancer (Sliva, 2006; Rios et al., 2012; Boh, 2013; Gill et al., 2016), antidiabetic (Chang et al., 2015; Ma et al., 2015; Pan et al., 2015), hepatoprotective (Ha do et al., 2013; Li et al., 2013; Wu et al., 2016), antiviral (especially anti-HIV activities; Min et al., 1998), and immunomodulating (Wang et al., 2014a,b). The anti-cancer of *G. lucidum* research is very popular in recent decades. It's believed that triterpenoids and polysaccharide are responsible for the anti-cancer effect. Besides, steroids and other kinds of compounds were also found in *G. lucidum*.

**Figure 1:**
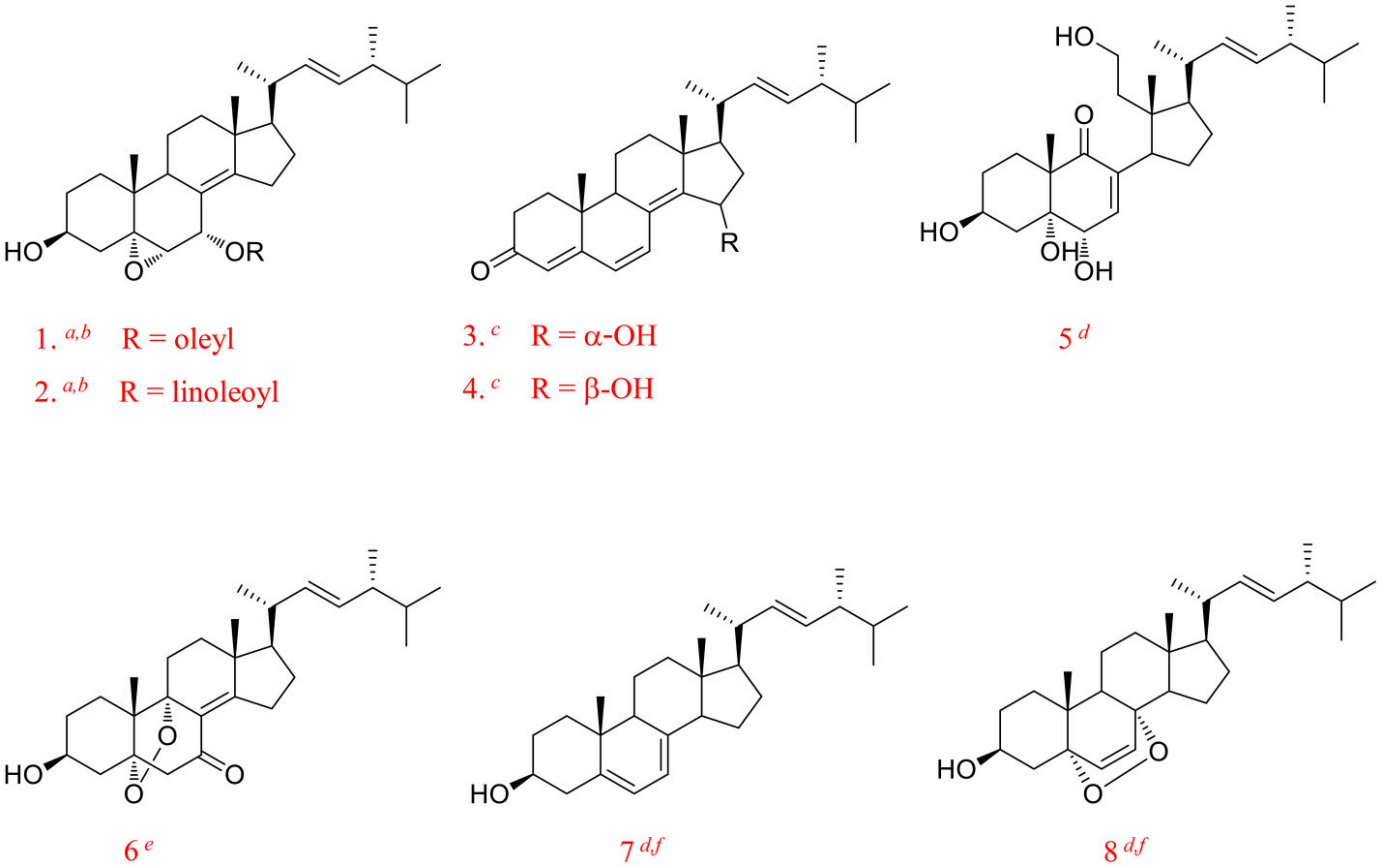
Some of the biologically active ergosterol derivatives from common edible mushrooms from literature. (1, Erinarol A; 2, Erinarol B; 3, Ganodermaside A; 4, Ganodermaside B; 5, (22*E*)-3β, 5α, 6α, 11-tetrahydroxy-9(11)-seco-ergosta-7, 22-dien-9-one; 6, Gargalols B; 7, Ergosterol; 8, Ergosterol peroxide; ^*a*^ PPAR transactivational effect; ^*b*^ Cytotoxicity; ^*c*^ Anti-aging; ^*d*^ Anti-inflammatory; ^*e*^ Suppressing osteoclast-forming; ^*f*^ Anti-cancer).

In recent years, we began to search bioactive secondary metabolites from edible and medicinal fungi and to study their mechanisms on chronic diseases. We had reported ergosterol peroxide from *G. lucidum* and ergosterol isolated from *Amauroderma rude* inhibiting cancer growth by up-regulating multiple tumor suppressors (Li et al., 2015, 2016). Steroids were a class of rich and important ingredients in edible and medicinal fungi since they play a pivotal role in maintaining normal structure and function of cell membranes and also act as precursors for the synthesis of metabolites like steroid hormones (Weete and Laseter, 1974; Mille-Lindblom et al., 2004; de Macedo-Silva et al., 2015). Sterols are reported to demonstrate immune-modulating (Bouic, 2002), anti-tumor (Yasukawa et al., 1994; Loza-Mejia and Salazar, 2015; Montserrat-de la Paz et al., 2015; Kiem et al., 2017), anti-inflammatory (Yasukawa et al., 1994; Kuo et al., 2011; Joy et al., 2017), anti-oxidative (Zhang et al., 2002), and other pharmacological activities (Kim and Ta, 2011; Zhu et al., 2014). Figure 1 showed some of the biologically active ergosterol derivatives reported from common edible fungi (Fan et al., 2006; Weng et al., 2010; Wu et al., 2011; Zhao, 2013; Li et al., 2014, 2015, 2016; Kikuchi et al., 2016). As ergosterol derivatives were multi-active and rich in fungi, they must be brought into focus during fungi research and development. However, current understanding of ergosterols composition in fungi is still not enough, especially on pharmacological mechanisms and structure-activity relationship.

In order to enrich the understanding of the diversity of ergosterols and to find more bioactive or higher active ergosterol derivatives, we carried out current work on *G. lucidum*. We now isolated 14 ergosterol derivatives, including one new compound, all of which showed anti-proliferative and anti-angiogenic activities *in vitro* in different levels. Herein, we described the structural elucidation and activity assay of these compounds. Their structure–activity relationships of cytotoxicity were also discussed. Besides that, the content of ergosterol in different parts of raw material and preparation of *G. lucidum* was also quantified.

## Materials and methods

### General experimental procedures

Analytical HPLC was performed on an Agilent 1200 with an Agilent DAD spectrophotometer (Agilent Technologies, Santa Clara, USA) and a YMC-Pack Pro C18 (5 μm, 4.6 × 250 mm, YMC Ltd., Japan). Preparative HPLC was performed on a Shimadzu LC-20A spectrophotometer and a YMC-Pack Pro C18 column (5 μm, 20 × 250 mm). NMR (1D and 2D) spectra were recorded by a Bruker AVANCE III 600 spectrometer. The ESI-MS spectra were measured on a 6430 Triple Quad mass spectrometer (Agilent Technologies, Santa Clara, USA). The HR-ESI-MS spectra were recorded using a Q-TOF mass spectrometer (Waters Corporation, Milford, USA). UV and IR data were measured using a JASCO V-550 UV/vis and a JASCO FT/IR-480 plus spectrometers (Jasco, Japan), respectively. Normal phase silica-gel (200–300 mesh) was purchased from Qingdao Haiyang Chemical Co., Ltd., Octadecylsilanized silica (ODS) gel (50 μm) was purchased from YMC Ltd. in Japan. The optical density was measured on a Tecan Infinite®200 PRO microplate reader (Tecan, Swiss).

### Macrofungi material

The fruiting bodies of *G. lucidum* had the same origination as that in our last published paper (Chen et al., 2017). They were all provided by Yuewei Edible Fungi Technology Co. Ltd., Guangzhou, China, and they originated from Dabie Mountain, Anhui, China. The voucher specimen (No. GL20160117) was deposited in State Key Laboratory of Applied Microbiology Southern China, Guangdong Institute of Microbiology.

### Extraction and isolation

The extraction and isolation procedure was similar to that of our last published paper (Chen et al., 2017). The dried fruiting bodies of *G. lucidum* (5.0 kg) were powdered and extracted with 95% ethanol (100 L × 2, liquid ratio 20:1) by heating-reflux to give a black crude extract (marked as GL, 116.2 g). As the extract was well dissolved in methanol-chloroform mixture, GL (100 g) was dissolved with methanol-chloroform (1:1) and mixed with silica gel. After solvent evaporating, the sample was added to an open ODS silica gel column and eluted with 35, 50, 75, and 100% methanol in sequence to give four fractions (marked as GL-1 to GL-4). GL-4 exhibited the strongest inhibitory effects on human tumor cells. Accordingly, GL-4 was selected for further isolation. Fraction GL-4 was subjected to an open silica-gel column chromatography eluted with cyclohexane-ethyl acetate-methanol (90:10:0, 80:20:0, 70:30:0, 50:50:0, 30:70:0.5, 0:100:1, 0:0:100) successively to afford 7 fractions marked as Fr. 4.1–Fr. 4.7. As the polarity of constituents in Frs. 4.5 and 4.6 were suitable for further isolation and purification based on TLC and HPLC analysis, Frs. 4.5 and 4.6 had been chosen for the subsequent isolation. Fr. 4.5 was further chose to purified on an ODS silica gel column and eluted with MeOH–H_2_O (50:50, 70:30, 80:20, 90:10, and 100:0) to produce 5 subfractions (Fr. 4.5.1–Fr. 4.5.5). Constituents in Frs. 4.5.4 and 4.5.3 had good peaks shape and resolution factor by HPLC analysis. Fr. 4.5.4 was purified on preparative HPLC eluted by 85% MeOH to afford compounds **2**, **3**, **4**, **5**, and **1**. Fr. 4.5.3 was further purified on prep-HPLC by 70% MeOH to give compounds **6**, **7**, and **8**. Fr. 4.6 was further subjected to a ODS column eluted with MeOH–H_2_O (50:50, 70:30, 80:20, 90:10, and 100:0) to afford five subfractions (Fr. 4.6.1–Fr. 4.6.5). Compound **12** was isolated from Fr. 4.6.5 by recrystallization. Similarly, Fr. 4.6.4 was suitable for further isolation. Fr. 4.6.4 was purified by preparative HPLC (MeOH–H_2_O, 90:10) to afford compounds **9**, **10**, **11**, and **13**. Compound **14** was isolated from Fr. 4.6.3 by prep-HPLC with 80% MeOH elution.

### Viability and cell death assay

Human breast carcinoma cells MDA-MB-231, human hepatocellular carcinoma cells HepG2, and human lung carcinoma cells A549, were purchased from ATCC, or were gifted by Professor Burton B. Yang from University of Toronto, Canada. The anti-proliferative effect of total extract (GL), fractions (GL1–GL4), and compounds **1**–**14** on tumor cell lines was evaluated by MTT assay (Franchi et al., 2012). Total extract (GL) and fractions (GL1–GL4) were evaluated for their anti-proliferative effect on three types of carcinoma cells (MDA-MB-231, HepG2, and A549). Compounds **1**–**14** were chosen to evaluate for their anti-proliferative effect against MDA-MB-231 and HepG2 cells. Hundred microliters of cells suspension (5 × 10^4^ cells/mL) was seeded into wells of a 96-well plate. After being incubated for 4 h, different concentrations of fractions/compounds were added onto the cells and incubated. After 48 h, 150 μL of 3-[4,5-dimethylthiazol-2-yl]-2,5-diphenyl tetrazolium bromide (MTT) solution (Sigma) was added to each well, and cells were kept being incubated for 4 h. After removing the MTT/medium, DMSO (100 μL) of was added to each well and was agitated at 60 rpm for 5 min. The optical density of the assay 96-well plate was read (λ = 540 nm) on a microplate reader.

### Anti-angiogenesis activity

HUVECs (KeyGEN BioTECH Co., Ltd, Jiangsu, China) were cultured in ECM (endothelial cell medium) with 5% (v/v) FBS, 1% (v/v) ECGS, and 1% penicillin-streptomycin (w/v) at 37°C in a cell incubator. The MTT assay was employed to assess the anti-angiogenic effect of the isolated compounds against HUVECs as described previously with mild modification (Liang et al., 2015; Nguyen et al., 2015). Briefly, HUVECs were seeded into a 96-well plate and incubated. Different concentrations of compounds **1–14** were added onto the cells after 4 h, and continued to be co-incubated for 48 h. Next, the cells were co-incubated with MTT (Sigma, St. Louis, MO, USA) to a final concentration of 0.05% for another 4 h. Then, replacing the MTT solution with 150 mL DMSO, and kept the plates being shaken for 3 min. The optical density of each well was measured on a microplate reader at λ = 570 nm.

### Statistical analysis

IC_50_-values were calculated and analyzed using SPSS with three replications, each carried out in triplicate. Data were expressed as mean ± SD and analyzed by one-way ANOVA followed by two-tailed Student's *t*-test. A difference was considered significant at the *P* < 0.05 or *P* < 0.01 level.

### 3D QSAR modeling

From the compounds, 15 compounds with accurate IC_50_s were chosen for 3D QSAR model establishment with Discovery Studio 3.1 (Luo et al., 2014; Sang et al., 2014). Firstly, the selected compounds were randomized into trainging set for model generation and test set for model validation, where training set divided 80% ratio. The remaining 20% (three compounds) for test set were compound 8, 14, and kaempferol. Then, the observed IC_50_s (μM) were converted to pIC_50_s, which were used as response variables. In model generation, force field was selected as CHARMm. Electrostatic potential were described by probing with a + 1e point charge and solvation were mimicked with distance dependent dielectric constant. Van der Waals potential was depicted by probing with a carbon atom of 1.73 Å in radius. The cut-off for electrostatic and steric energies was 30 kcal/mol. Others were kept as default.

### Quantification of ergosterol in different batches of raw *G. lucidum* and products made from *G. lucidum*

#### HPLC conditions

Analyses were performed on an Agilent 1200 HPLC instrument, equipped with a G1311A pump and a G1314A UV detector. The chromatographic separation was achieved on an Agilent prep-C_18_ analytical column (4.6 × 250 mm, 5 μm, Agilent) eluted with 95% methanol at a flow rate of 1.0 mL/min, maintained at 35°C. The choice UV detection wavelength was 282 nm. All injection volumes were 20 μL.

#### Preparation of reference solution

Ergosterol was accurately weighed and dissolved in methanol at 2.00 μg/mL. The stock solution was stored at 4°C protected against bright light.

#### Preparation of test solution

To 1.00 g of the powdered raw material of *G. lucidum* add 50.0 mL 95% ethanol. Weigh and heat under a reflux condenser for 1 h. Allow to cool. Make up the weight loss with the same solvent, mix well, and filter. Transfer the solution into a 50.0 mL volumetric flask and bring to volume with methanol. To 0.40 g of the spore oil or the spore extract of *G. lucidum* transfer to a 10.0 mL volumetric flask, add 95% ethanol to the scale line and mix well. All the test solution was filtered respectively through a 0.45 μm membrane prior to an injection into HPLC system. Carry out the assay protected against bright light.

## Results and discussion

### Extraction and preparation of the sterols/lipids enriched fraction

**Figure 2:**
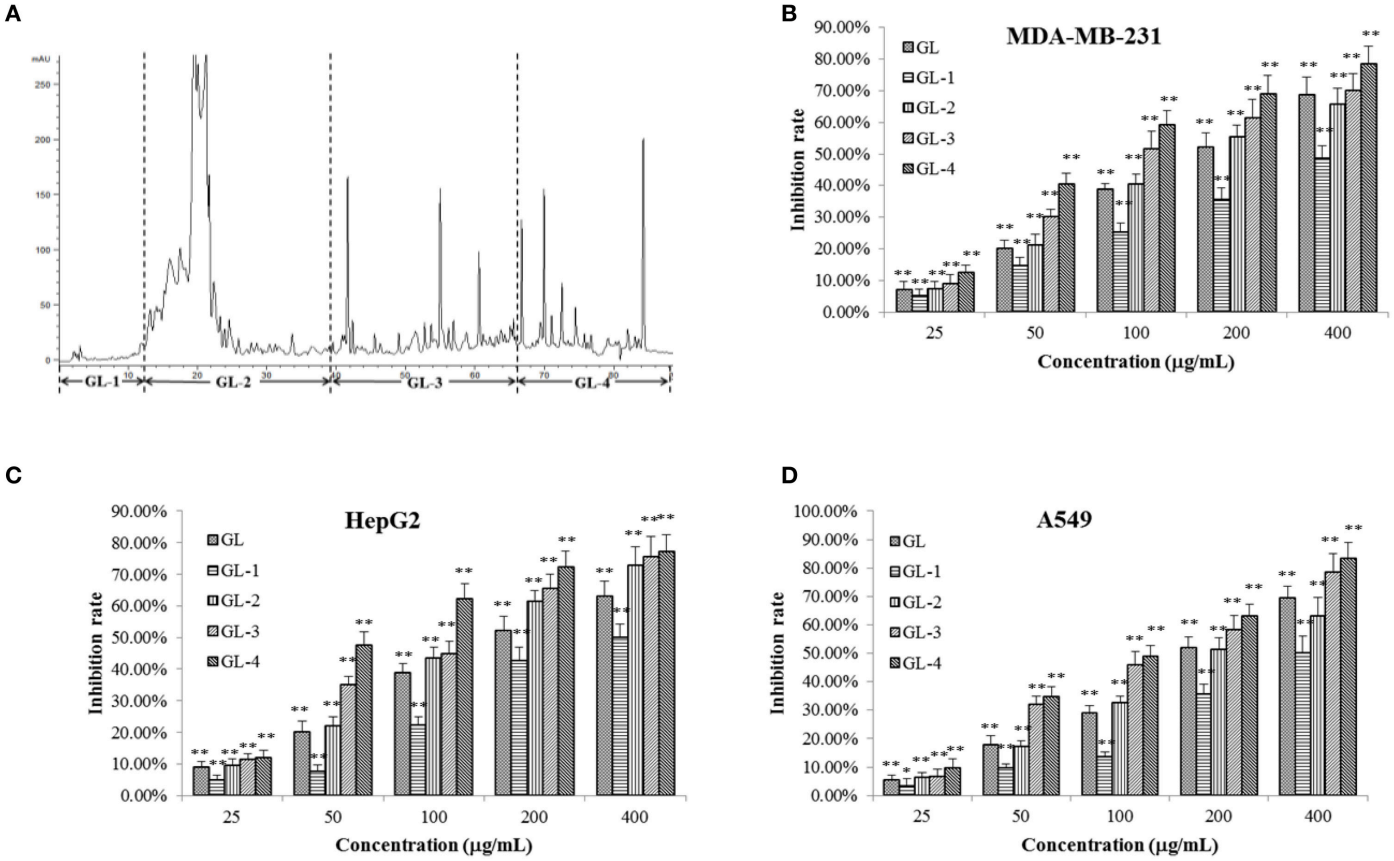
Chromatrogram of the GL extract **(A)** at 254 nm and anti-proliferative effects of its fractions against MDA-MB-231 **(B)**, HepG2 **(C)**, and A549 **(D)** cells. The asterisms indicate that the values were significantly different from the controls (^*^p < 0.05, ^**^p < 0.01).

In brief, the dried fruiting bodies of *G. lucidum* were powdered and extracted with 95% ethanol by heating-reflux to give a black crude extract (marked as GL). GL was dissolved with methanol-chloroform and mixed with silica gel. After solvent evaporating, the sample was loaded to an open ODS silica-gel column chromatograph eluted by gradient 30, 50, 75, and 100% methanol in sequence to give four fractions, which were marked as: water soluble fraction (marked as GL-1, 25.8 g), ganoderic acid enriched fraction (GL-2, 19.1 g), ganoderol enriched fraction (GL-3, 6.5 g), and lipids enriched fraction (GL-4, 34.8 g). Figure 2A showed the constituents complexity of GL-1–GL-4 by HPLC analysis at λ 254 nm.

An MTT assay was employed to assess the inhibitory effects of GL samples on MDA-MB-231, HepG2, and A549 cells (Figures 2B–D). It was observed that GL samples (*p* < 0.05) inhibited the proliferation of three tumor cell lines compared to the blank control. For all the three tumor cells, within the concentration of 25–400 μg/mL, all samples inhibited the proliferation of three types of human cancer cells concentration-dependently. All samples possessed similar capability to inhibit the growth of MDA-MB-231, HepG2, and A549 cells. Significant reductions (*p* < 0.01) in cell viabilities were observed under the interference of GL, GL-2, GL-3, and GL-4 fractions except GL-1. This suggested that the active constituents may concentrate in the GL2, GL3, and GL4 fractions. GF-4 fraction displayed a much higher inhibition rate (*p* < 0.01) than GF. Besides, GF-4 also showed higher inhibition rate (*p* < 0.05) than GL-2 and GL-3, indicating GL-4 were the best bioactive fraction. Since the total triterpenoids fraction (GL-2 and GL-3) has been studied in our previous research (Chen et al., 2017), as well as the lipids enriched fraction (GL-4) showed higher inhibitory effects on tumor cells than other fractions. Therefore, GL-4 was selected for further research in this paper.

### Identification of compounds

Bioassay-guided fraction (GL-4) of *G. lucidum* extract (GL) led to 14 ergostane-type sterols. Their chemical structures were established based on comprehensive spectroscopic analyses or comparison of mass and NMR spectroscopic data with those literatures reported.

**Figure 3:**
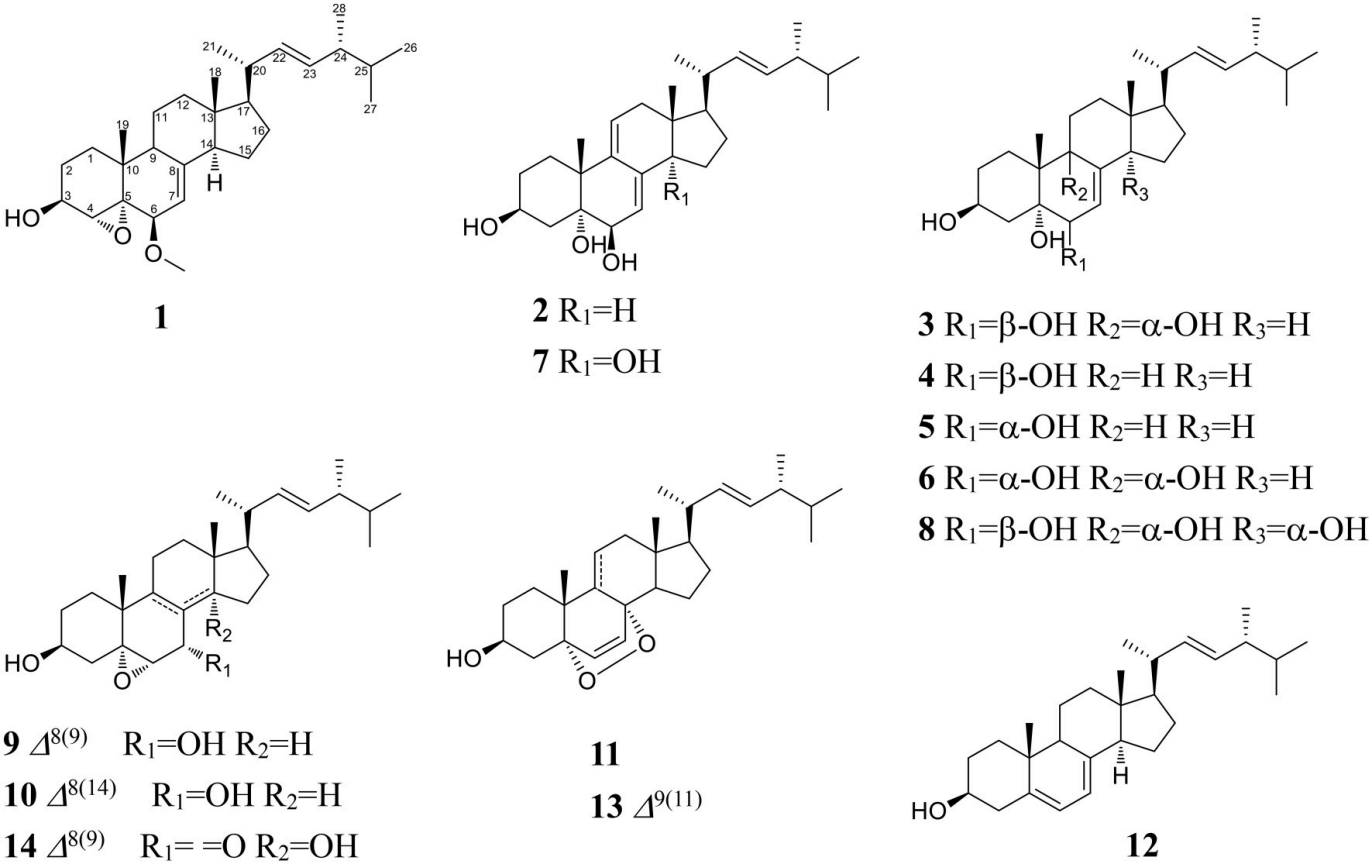
Structures of compounds **1–14**.

**Figure 4:**
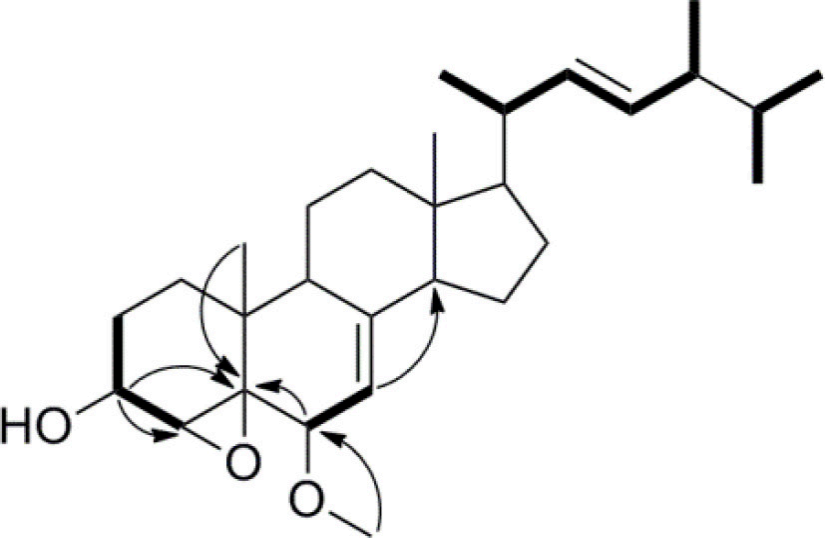
Key HMBC and ^1^H-^1^H COSY correlations of compound **1**.

Compound **1** was isolated as a white amorphous powder. Its molecular formula of C_29_H_46_O_3_ was determined based on the HR-ESI-MS *m/z* 443.3505 [M + H]^+^ (calcd for C_29_H_47_O_3_, 443.3525), indicating the presence of seven degrees of unsaturation in the molecule. A strong absorption (λ_max_) at 245 nm in UV spectrum declaring a conjugated diene system existed in the chemical structure. The ^13^C NMR together with DEPT-135 spectra presented 29 carbons, which attributed to seven methyls inclusive of one methoxyl, six methylenes, 12 methines (three oxygenated), and four quaternary carbons. Typical signals of a sterol, such as, a side-chain olefine (*δ*_H_ 5.15–5.19, *m*; 5.19–5.22, *m*), four doublet methyls (*δ*_H_ 0.82, *d*, 6.6; 0.85, *d*, 6.6; 0.92, *d*, 6.6; 1.02, *d*, 6.0), and two singlet methyls (*δ*_H_ 0.63, *s*; 1.30, *s*) were observed from the ^1^H-NMR spectrum. Comparison of the ^1^H- and ^13^C NMR spectroscopic data of compound **1** with those of (22*E*)-4*α*,5*α*-epoxyergosta-7,22-diene-3*β*,6*β*-diol, an ergostane-type steriod with molecular formula C_28_H_44_O_3_, isolated from another mushroom *Pleurotus eryngii* (Kikuchi et al., 2016), the ^13^C-NMR data were almost the same except for an obvious different chemical shift of C-6. The downshift of C-6 from *δ*_H_ 72.0 to 80.9 suggested that the hydroxyl at C-6 was methylated. The location of the methoxyl at C-6 could also be verified by HMBC correlation from the methoxyl signal at *δ*_H_ 3.38 to *δ*_C_ 80.9 (C-6). Thus, the structure of **1** was determined to be (22*E*)-4*α*, 5*α*-epoxy-6*β*-methoxyergosta-7, 22-diene-3*β*-ol (Figure 3). Compound **1** was a new compound has not been reported. The complete assignment of the protons and carbons were summarized in Table 1. The key HMBC and ^1^H-^1^H COSY correlations were also shown in Figure 4.

**Table 1:** ^1^H and ^13^C NMR spectral data of compound **1** in CDCl_3_(^1^H for 600 MHz, ^13^C for 150 MHz).

**No**.	***δ*_H_**	***δ*_C_**
1	1.34~1.36 (1H, m) *^a^*, 1.26~1.28 (1H, m)	29.8
2	1.94~1.96 (1H, m), 1.32~1.35 (1H, m)	26.8
3	4.05 (1H, brt, 8.4)	65.7
4	3.15 (1H, s)	62.7
5	–	66.1
6	3.01 (1H, br.s)	80.9
7	5.41~5.43 (1H, m)	115.4
8	–	144.2
9	2.00~2.03 (1H, m)	45.2
10	–	33.6
11	1.53~1.56 (1H, m), 1.41~1.43 (1H, m)	21.4
12	2.06~2.08 (1H, m), 1.33~1.35 (1H, m)	39.0
13	–	43.7
14	1.91~1.93 (1H, m)	54.8
15	1.55~1.59 (2H, m)	22.9
16	1.73~1.76 (1H, m), 1.28~1.31 (1H, m)	27.9
17	1.30~1.33 (1H, m)	56.0
18	0.63 (3H, s)	12.3
19	1.28 (3H, s)	18.9
20	2.02~2.06 (1H, m)	40.5
21	1.02 (3H, d, 6.0)	21.0
22	5.15~5.19 (1H, m)	135.5
23	5.19~5.22 (1H, m)	132.2
24	1.80~1.83 (1H, m)	42.7
25	1.40~1.43 (1H, m)	33.2
26	0.82 (3H, d, 6.6)	19.8
27	0.85 (3H, d, 6.6)	20.0
28	0.92 (3H, d, 6.6)	17.9
−OCH_3_	3.38 (3H, s)	58.3

a *Means multiplet or overlapped with other signals*.

(3*β*, 5*α*, 6*β*, 22*E*)-ergosta-7, 9 (11), 22-triene-3, 5, 6-triol (**2**): a white powder; Its negative-ion ESI-MS (*m/z*) at 427 [M–H]^−^, 855 [2M–H] ^−^ indicated the molecular weight was 428. ^1^H-NMR (600 MHz, CDCl_3_): *δ*_H_ 4.10–4.12 (1H, *m*, H-3), 3.80 (1H, br.*s*, H-6), 5.45 (1H, *d*, 6.0, H-7), 5.75 (1H, *d*, 6.6, H-11), 0.62 (3H, *s*, H-18), 1.28 (3H, *s*, H-19), 1.01 (3H, *d*, 6.6, H-21), 5.16 (1H, *dd*, 15.0, 7.2, H-22), 5.26 (1H, *dd*, 15.0, 7.2, H-23), 0.81 (3H, *d*, 6.6, H-26), 0.83 (3H, *d*, 6.6, H-27) and 0.91 (3H, *d*, 6.6, H-28). ^13^C-NMR (150 MHz, CDCl_3_): *δ*_C_ 29.7 (C-1), 31.0 (C-2), 67.7 (C-3), 42.2 (C-4), 75.3 (C-5), 74.0 (C-6), 118.2 (C-7), 138.9 (C-8), 140.0 (C-9), 40.7 (C-10), 126.4 (C-11), 42.6 (C-12), 42.6 (C-13), 51.5 (C-14), 23.2 (C-15), 28.7 (C-16), 56.0 (C-17), 11.5 (C-18), 26.3 (C-19), 40.3 (C-20), 20.8 (C-21), 135.2 (C-22), 132.3 (C-23), 42.8 (C-24), 33.1 (C-25), 19.7 (C-26), 20.0 (C-27), and 17.6 (C-28). Based on these ^1^H- and ^13^C-NMR data, compound **2** was identified as (3*β*, 5*α*, 6*β*, 22*E*)-ergosta-7, 9 (11), 22-triene-3, 5, 6-triol by comparison with the data reported previously (Ishizuka et al., 1997).

(3*β*, 5*α*, 6*β*, 9*α*, 22*E*)-ergosta-7, 22-diene-3, 5, 6, 9-tetrol (**3**): a white powder; Its molecular weight was 446 by negative-ion ESI-MS (*m/z*) at 445 [M–H]^−^, 891 [2M–H] ^−^. ^1^H-NMR (600 MHz, CDCl_3_): *δ*_H_ 3.41–3.43 (1H, *m*, H-3), 3.55 (1H, br.*s*, H-6), 5.78 (1H, *d*, 6.0, H-7), 0.60 (3H, *s*, H-18), 1.11 (3H, *s*, H-19), 1.00 (3H, *d*, 6.6, H-21), 5.13 (1H, *dd*, 15.0, 7.2, H-22), 5.25 (1H, *dd*, 15.0, 7.2, H-23), 0.82 (6H, *d*, 6.6, H-26, 27) and 0.92 (3H, *d*, 6.6, H-28). ^13^C-NMR (150 MHz, CDCl_3_): *δ*_C_ 29.3 (C-1), 31.6 (C-2), 67.5 (C-3), 40.8 (C-4), 75.5 (C-5), 72.3 (C-6), 121.3 (C-7), 141.7 (C-8), 78.6 (C-9), 41.3 (C-10), 28.5 (C-11), 35.6 (C-12), 44.5 (C-13), 50.9 (C-14), 23.4 (C-15), 28.5 (C-16), 57.1 (C-17), 12.1 (C-18), 22.5 (C-19), 40.4 (C-20), 21.4 (C-21), 135.4 (C-22), 132.3 (C-23), 42.8 (C-24), 33.5 (C-25), 19.9 (C-26), 20.2 (C-27), and 17.7 (C-28). The ^1^H- and ^13^C-NMR data for compound **3** were found to be in agreement with published data for (3*β*, 5*α*, 6*β*, 9*α*, 22*E*)-ergosta-7, 22-diene-3, 5, 6, 9-tetrol (Zou et al., 2013).

(3*β*, 5*α*, 6*β*, 22*E*)-ergosta-7, 22-diene-3, 5, 6-triol (**4**): a white powder; The negative-ion ESI-MS (*m/z*) at 429 [M–H]^−^, 859 [2M–H] ^−^, indicated a molecular weight of 430. ^1^H-NMR (600 MHz, CDCl_3_): *δ*_H_ 4.05–4.07 (1H, *m*, H-3), 3.60 (1H, br.*s*, H-6), 5.38 (1H, *d*, 6.0, H-7), 0.60 (3H, *s*, H-18), 1.08 (3H, *s*, H-19), 1.00 (3H, *d*, 6.6, H-21), 5.13 (1H, *dd*, 15.0, 7.2, H-22), 5.25 (1H, *dd*, 15.0, 7.2, H-23), 0.82 (6H, *d*, 6.6, H-26, 27) and 0.92 (3H, *d*, 6.6, H-28). ^13^C-NMR (150 MHz, CDCl_3_): *δ*_C_ 33.0 (C-1), 31.1 (C-2), 67.5 (C-3), 39.6 (C-4), 75.9 (C-5), 73.8 (C-6), 117.6 (C-7), 143.9 (C-8), 43.6 (C-9), 37.2 (C-10), 22.1 (C-11), 39.2 (C-12), 43.7 (C-13), 54.9 (C-14), 22.8 (C-15), 28.0 (C-16), 56.1 (C-17), 12.1 (C-18), 18.9 (C-19), 40.4 (C-20), 21.4 (C-21), 135.4 (C-22), 132.3 (C-23), 42.8 (C-24), 33.3 (C-25), 19.9 (C-26), 20.2 (C-27), and 17.7 (C-28). Thus, compound **4** was identified as (3*β*, 5*α*, 6*β*, 22*E*)-ergosta-7, 22-diene-3, 5, 6-triol by comparison the ^1^H-NMR and ^13^C-NMR data with the data reported previously (Kawagishi et al., 1988).

(3*β*, 5*α*, 6*α*, 22*E*)-ergosta-7, 22-diene-3, 5, 6-triol (**5**): a white powder; The molecular weight of 430 was also determined by negative-ion ESI-MS (*m/z*) at 429 [M–H]^−^, 859 [2M–H]^−^. ^1^H-NMR (600 MHz, CDCl_3_): *δ*_H_ 3.98–4.02 (1H, *m*, H-3), 3.94 (1H, br.*s*, H-6), 5.00 (1H, *d*, 6.0, H-7), 0.59 (3H, *s*, H-18), 1.05 (3H, *s*, H-19), 1.26 (3H, *d*, 6.0, H-21), 5.13 (1H, *dd*, 15.0, 7.2, H-22), 5.25 (1H, *dd*, 15.0, 7.2, H-23), 0.82 (6H, *d*, 6.6, H-26), 0.88 (6H, *d*, 6.6, H-27) and 0.95 (3H, *d*, 6.6 Hz, H-28). ^13^C-NMR (150 MHz, CDCl_3_): *δ*_C_ 39.6 (C-1), 32.2 (C-2), 67.0 (C-3), 32.5 (C-4), 75.8 (C-5), 70.3 (C-6), 121.3 (C-7), 140.8 (C-8), 33.2 (C-9), 39.2 (C-10), 21.5 (C-11), 41.0 (C-12), 43.7 (C-13), 54.9 (C-14), 22.8 (C-15), 28.5 (C-16), 56.0 (C-17), 12.2 (C-18), 17.8 (C-19), 40.4 (C-20), 21.4 (C-21), 135.4 (C-22), 132.3 (C-23), 42.8 (C-24), 40.7 (C-25), 21.3 (C-26), 20.2 (C-27), and 19.7 (C-28). Compound **5** was identified as (3*β*, 5*α*, 6*α*, 22*E*)-ergosta-7, 22-diene-3, 5, 6-triol by comparison with the data reported previously (Chen et al., 1991).

(3*β*, 5*α*, 6*α*, 9*α*, 22*E*)-ergosta-7, 22-diene-3, 5, 6, 9-tetrol (**6**): a white powder; The negative-ion ESI-MS (*m/z*) displayed quasi-molecular ion peaks at 445 [M–H]^−^, 891 [2M–H]^−^, thus its molecular weight was 446. ^1^H-NMR (600 MHz, CDCl_3_): *δ*_H_ 4.00–4.03 (1H, *m*, H-3), 3.93 (1H, br.*s*, H-6), 5.10 (1H, *d*, 6.0, H-7), 0.60 (3H, *s*, H-18), 1.07 (3H, *s*, H-19), 1.02 (3H, *d*, 6.6, H-21), 5.15 (1H, *dd*, 15.0, 7.2, H-22), 5.25 (1H, *dd*, 15.0, 7.2, H-23), 0.82 (6H, *d*, 6.6, H-26, 27) and 0.92 (3H, *d*, 6.6, H-28). ^13^C-NMR (150 MHz, CDCl_3_): *δ*_C_ 26.5 (C-1), 30.6 (C-2), 67.4 (C-3), 40.2 (C-4), 77.0 (C-5), 70.3 (C-6), 120.3 (C-7), 142.5 (C-8), 74.6 (C-9), 41.1 (C-10), 28.4 (C-11), 35.4 (C-12), 43.9 (C-13), 50.7 (C-14), 23.0 (C-15), 28.5 (C-16), 56.1 (C-17), 11.9 (C-18), 20.5 (C-19), 40.4 (C-20), 21.4 (C-21), 135.4 (C-22), 132.5 (C-23), 42.8 (C-24), 33.3 (C-25), 19.9 (C-26), 20.2 (C-27), and 17.7 (C-28). Based on these ^1^H-NMR and ^13^C-NMR data, compound **6** was identified as (3*β*, 5*α*, 6*α*, 9*α*, 22*E*)-ergosta-7, 22-diene-3, 5, 6, 9-tetrol by comparison with the data reported previously (Yaoita et al., 1998).

(3*β*, 5*α*, 6*β*, 14*α*, 22*E*)-ergosta-7, 9 (11), 22-triene-3, 5, 6, 14-tetrol (**7**): a white powder; Its negative-ion ESI-MS (*m/z*) 443 [M–H]^−^, 887 [2M–H] ^−^ indicated a molecular weight of 444. ^1^H-NMR (600 MHz, CDCl_3_): *δ*_H_ 4.04–4.08 (1H, *m*, H-3), 3.80 (1H, br.*s*, H-6), 5.85 (1H, *d*, 6.0, H-7), 5.55 (1H, *d*, 6.6, H-11), 0.80 (3H, *s*, H-18), 1.22 (3H, *s*, H-19), 1.01 (3H, *d*, 6.6, H-21), 5.16 (1H, *dd*, 15.0, 7.2, H-22), 5.26 (1H, *dd*, 15.0, 7.2, H-23), 0.81 (3H, *d*, 6.6, H-26), 0.83 (3H, *d*, 6.6, H-27) and 0.91 (3H, *d*, 6.6, H-28). ^13^C-NMR (150 MHz, CDCl_3_): *δ*_C_ 31.3 (C-1), 31.0 (C-2), 67.5 (C-3), 38.3 (C-4), 75.1 (C-5), 73.5 (C-6), 120.2 (C-7), 137.3 (C-8), 138.2 (C-9), 39.4 (C-10), 124.2 (C-11), 37.8 (C-12), 46.0 (C-13), 81.5 (C-14), 35.2 (C-15), 23.8 (C-16), 50.0 (C-17), 16.5 (C-18), 24.3 (C-19), 40.0 (C-20), 22.3 (C-21), 134.5 (C-22), 133.3 (C-23), 43.1 (C-24), 33.0 (C-25), 19.7 (C-26), 20.0 (C-27), and 17.6 (C-28). Based on these ^1^H-NMR and ^13^C-NMR data, compound **7** was identified as (3*β*, 5*α*, 6*β*, 14*α*, 22*E*)-ergosta-7, 9 (11), 22-triene-3, 5, 6, 14-tetrol by comparison with the data reported previously (Zang et al., 2013).

(3*β*, 5*α*, 6*β*, 9*α*, 14*α*, 22*E*)-ergosta-7, 22-diene-3, 5, 6, 9, 14-pentol (**8**): a white powder; Its negative-ion ESI-MS (*m/z*) displayed quasi-molecular ion peaks at 461 [M–H]^−^, 921 [2M–H] ^−^, indicating a molecular weight of 462. ^1^H-NMR (600 MHz, C_5_D_5_N): *δ*_H_ 4.84–4.87 (1H, *m*, H-3), 4.57 (1H, br.*s*, H-6), 7.10 (1H, *d*, 6.0, H-7), 3.26–3.29 (1H, *m*, H-15), 1.41 (3H, *s*, H-18), 1.62 (3H, *s*, H-19), 1.24 (3H, *d*, 6.6, H-21), 5.75 (1H, *dd*, 15.0, 7.2, H-22), 5.42 (1H, *dd*, 15.0, 7.2, H-23), 0.92 (6H, *d*, 6.6, H-26, 27) and 1.05 (3H, *d*, 6.6, H-28). ^13^C-NMR (150 MHz, C_5_D_5_N): *δ*_C_ 27.9 (C-1), 32.7 (C-2), 67.5 (C-3), 42.2 (C-4), 78.6 (C-5), 74.0 (C-6), 123.9 (C-7), 145.0 (C-8), 75.9 (C-9), 41.9 (C-10), 28.5 (C-11), 38.1 (C-12), 48.5 (C-13), 84.7 (C-14), 42.2 (C-15), 28.4 (C-16), 56.6 (C-17), 17.5 (C-18), 22.2 (C-19), 39.9 (C-20), 23.0 (C-21), 135.9 (C-22), 132.8 (C-23), 43.5 (C-24), 33.5 (C-25), 19.9 (C-26), 20.2 (C-27), and 18.1 (C-28). Based on these ^1^H-NMR and ^13^C-NMR data, compound **8** was identified as (3*β*, 5*α*, 6*β*, 9*α*, 14*α*, 22*E*)-ergosta-7, 22-diene-3, 5, 6, 9, 14-pentol by comparison with the data reported previously (Zhang et al., 2008).

(3*β*, 5*α*, 6*α*, 7*α*, 22*E*)-5, 6-epoxy-ergosta-8, 22-diene-3, 7-diol (**9**): a white powder; Its negative-ion ESI-MS (*m/z*) displayed quasi-molecular ion peaks at 427 [M–H]^−^, 855 [2M–H]^−^, indicating a molecular weight of 428. ^1^H-NMR (600 MHz, CDCl_3_): *δ*_H_ 3.93–3.96 (1H, *m*, H-3), 3.31 (1H, br.*s*, H-6), 4.25 (1H, *d*, 6.0, H-7), 0.60 (3H, *s*, H-18), 1.15 (3H, *s*, H-19), 1.01 (3H, *d*, 6.6, H-21), 5.16 (1H, *dd*, 15.0, 7.2, H-22), 5.25 (1H, *dd*, 15.0, 7.2, H-23), 0.81 (3H, *d*, 6.6, H-26), 0.83 (3H, *d*, 6.6, H-27) and 0.91 (3H, *d*, 6.6, H-28). ^13^C-NMR (150 MHz, CDCl_3_): *δ*_C_ 30.3 (C-1), 31.0 (C-2), 68.6 (C-3), 39.3 (C-4), 65.7 (C-5), 62.6 (C-6), 67.1 (C-7), 127.0 (C-8), 134.2 (C-9), 38.2 (C-10), 23.4 (C-11), 35.8 (C-12), 42.0 (C-13), 49.6 (C-14), 23.8 (C-15), 28.8 (C-16), 53.7 (C-17), 11.5 (C-18), 22.8 (C-19), 40.4 (C-20), 20.9 (C-21), 135.5 (C-22), 132.3 (C-23), 42.8 (C-24), 33.0 (C-25), 19.7 (C-26), 20.0 (C-27), and 17.6 (C-28). Compound **9** was identified as (3*β*, 5*α*, 6*α*, 7*α*, 22*E*)-5, 6-epoxy-ergosta-8, 22-diene-3, 7-diol (Della Greca et al., 1993).

(3*β*, 5*α*, 6*α*, 7*α*, 22*E*)-5, 6-epoxy-ergosta-8 (14), 22-diene-3, 7-diol (**10**): a white powder; Its molecular weight was also 428 by negative-ion ESI-MS (*m/z*) 427 [M–H]^−^, 855 [2M–H]^−^. ^1^H-NMR (600 MHz, CDCl_3_): *δ*_H_ 3.91–3.93 (1H, *m*, H-3), 3.12 (1H, br.*s*, H-6), 4.40 (1H, *d*, 6.0, H-7), 0.86 (6H, *s*, H-18, 19), 1.01 (3H, *d*, 6.6, H-21), 5.16 (1H, *dd*, 15.0, 7.2, H-22), 5.25 (1H, *dd*, 15.0, 7.2, H-23), 0.81 (3H, *d*, 6.6, H-26), 0.83 (3H, *d*, 6.6, H-27) and 0.91 (3H, *d*, 6.0, H-28). ^13^C-NMR (150 MHz, CDCl_3_): *δ*_C_ 32.3 (C-1), 31.0 (C-2), 68.6 (C-3), 39.5 (C-4), 67.7 (C-5), 61.3 (C-6), 65.1 (C-7), 125.0 (C-8), 38.8 (C-9), 35.8 (C-10), 19.0 (C-11), 36.6 (C-12), 43.0 (C-13), 152.6 (C-14), 25.0 (C-15), 27.1 (C-16), 56.7 (C-17), 18.1 (C-18), 16.5 (C-19), 39.4 (C-20), 21.0 (C-21), 135.5 (C-22), 132.3 (C-23), 42.8 (C-24), 33.0 (C-25), 19.7 (C-26), 20.0 (C-27), and 17.6 (C-28). The ^1^H-NMR and ^13^C-NMR data of compound **10** were in agreement with previously published data for (3*β*, 5*α*, 6*α*, 7*α*, 22*E*)-5, 6-epoxy-ergosta-8 (14), 22-diene-3, 7-diol (Della Greca et al., 1993).

(3*β*, 5*α*, 8*α*, 22*E*)-5, 8-epidioxy-ergosta-6, 22-dien-3-ol (**11**): a white powder; Its molecular weight was the same as compounds **9** and **10**. ^1^H-NMR (600 MHz, CDCl_3_): *δ*_H_ 3.91–3.93 (1H, *m*, H-3), 6.24 (1H, *d*, 8.4, H-6), 6.50 (1H, *d*, 8.4, H-7), 0.81 (3H, *s*, H-18), 0.88 (3H, *s*, H-19), 1.01 (3H, *d*, 6.6, H-21), 5.16 (1H, *dd*, 15.0, 7.2, H-22), 5.25 (1H, *dd*, 15.0, 7.2, H-23), 0.81 (3H, *d*, 6.6, H-26), 0.83 (3H, *d*, 6.6, H-27) and 0.91 (3H, *d*, 6.6, H-28). ^13^C-NMR (150 MHz, CDCl_3_): *δ*_C_ 34.7 (C-1), 30.1 (C-2), 66.6 (C-3), 37.0 (C-4), 82.2 (C-5), 135.4 (C-6), 130.8 (C-7), 79.4 (C-8), 51.1 (C-9), 36.9 (C-10), 23.4 (C-11), 39.4 (C-12), 44.6 (C-13), 51.6 (C-14), 20.6 (C-15), 28.6 (C-16), 56.2 (C-17), 12.9 (C-18), 18.2 (C-19), 39.7 (C-20), 21.0 (C-21), 135.2 (C-22), 132.3 (C-23), 42.8 (C-24), 33.0 (C-25), 19.7 (C-26), 20.0 (C-27), and 17.6 (C-28). Compound **11** was identified as (3*β*, 5*α*, 8*α*, 22*E*)-5, 8-epidioxy-ergosta-6, 22-dien-3-ol by comparison with the data reported previously (Liu et al., 2007).

(3*β*, 22*E*)-ergosta-5, 7, 22-trien-3-ol (**12**): Colorless crystal; Its molecular weight was 396 also by negative-ion ESI-MS. ^1^H-NMR (600 MHz, CDCl_3_): *δ*_H_ 3.63–3.66 (1H, *m*, H-3), 5.60 (1H, *dd*, 6.6, 6.0, H-6), 5.40–5.44 (1H, *m*, H-7), 0.62 (3H, *s*, H-18), 0.96 (3H, *s*, H-19), 1.04 (3H, *d*, 6.6, H-21), 5.18 (1H, *dd*, 15.6, 7.2, H-22), 5.22 (1H, *dd*, 15.6, 7.2, H-23), 0.85 (6H, *d*, 6.6, H-26, 27) and 0.92 (3H, *d*, 6.6, H-28). ^13^C-NMR (150 MHz, CDCl_3_): *δ*_C_ 39.1 (C-1), 32.0 (C-2), 70.5 (C-3), 40.8 (C-4), 139.8 (C-5), 119.6 (C-6), 116.3 (C-7), 141.4 (C-8), 46.2 (C-9), 37.0 (C-10), 21.3 (C-11), 38.4 (C-12), 42.8 (C-13), 54.6 (C-14), 23.0 (C-15), 28.3 (C-16), 55.5 (C-17), 12.1 (C-18), 16.3 (C-19), 40.2 (C-20), 21.3 (C-21), 135.5 (C-22), 132.3 (C-23), 43.1 (C-24), 33.0 (C-25), 19.7 (C-26), 20.0 (C-27), and 17.6 (C-28). The ^1^H-NMR and ^13^C-NMR data for compound **12** were in agreement with previously published data for ergosterol (Kang et al., 2003).

(3*β*, 5*α*, 8*α*, 22*E*)-5, 8-epidioxy-ergosta-6, 9 (11), 22-trien-3-ol (**13**): a white powder; Its negative-ion ESI-MS (*m/z*) displayed quasi-molecular ion peaks at 425 [M–H]^−^, 851 [2M–H]^−^, indicating a molecular weight of 426. ^1^H-NMR (600 MHz, CDCl_3_): *δ*_H_ 3.91–3.93 (1H, *m*, H-3), 6.24 (1H, *d*, 8.4, H-6), 6.50 (1H, *d*, 8.4, H-7), 0.81 (3H, *s*, H-18), 0.88 (3H, *s*, H-19), 1.01 (3H, *d*, 6.6, H-21), 5.16 (1H, *dd*, 15.0, 7.2, H-22), 5.25 (1H, *dd*, 15.0, 7.2, H-23), 0.81 (3H, *d*, 6.6, H-26), 0.83 (3H, *d*, 6.6, H-27) and 0.91 (3H, *d*, 6.6, H-28). ^13^C-NMR (150 MHz, CDCl_3_): *δ*_C_ 34.7 (C-1), 30.1 (C-2), 66.6 (C-3), 37.0 (C-4), 82.2 (C-5), 136.4 (C-6), 130.9 (C-7), 78.8 (C-8), 144.2 (C-9), 38.6 (C-10), 119.2 (C-11), 41.5 (C-12), 43.9 (C-13), 48.9 (C-14), 21.6 (C-15), 28.9 (C-16), 56.2 (C-17), 13.2 (C-18), 25.7 (C-19), 40.0 (C-20), 21.0 (C-21), 135.5 (C-22), 132.3 (C-23), 42.8 (C-24), 33.4 (C-25), 19.7 (C-26), 20.0 (C-27), and 17.6 (C-28). Compound **13** was identified as (3*β*, 5*α*, 8*α*, 22*E*)-5, 8-epidioxy-ergosta-6, 22-dien-3-ol by comparison with the data reported (Ma et al., 1994).

(3*β*, 5*α*, 6*α*, 14*α*, 22*E*)-3, 14-dihydroxy-5, 6-epoxy-ergosta-8, 22-dien-7-one (**14**): a white powder; The molecular weight of 442 was determined by negative-ion ESI-MS (*m/z*) 441 [M–H]^−^, 883 [2M–H] ^−^. ^1^H-NMR (600MHz, CDCl_3_): *δ*_H_ 3.94–3.96 (1H, *m*, H-3), 3.35 (1H, *s*, H-6), 0.92 (3H, *s*, H-18), 1.21 (3H, *s*, H-19), 1.01 (3H, *d*, 6.6, H-21), 5.16 (1H, *dd*, 15.0, 7.2, H-22), 5.25 (1H, *dd*, 15.0, 7.2, H-23), 0.80 (3H, *d*, 6.6, H-26), 0.83 (3H, *d*, 6.6, H-27) and 0.88 (3H, *d*, 6.6, H-28). ^13^C-NMR (150 MHz, CDCl_3_): *δ*_C_ 28.9 (C-1), 30.1 (C-2), 68.6 (C-3), 38.3 (C-4), 65.6 (C-5), 62.3 (C-6), 200.2 (C-7), 133.1 (C-8), 157.0 (C-9), 40.7 (C-10), 23.0 (C-11), 29.8 (C-12), 44.9 (C-13), 80.9 (C-14), 35.4 (C-15), 25.9 (C-16), 44.8 (C-17), 16.3 (C-18), 23.0 (C-19), 39.8 (C-20), 21.0 (C-21), 135.0 (C-22), 132.7 (C-23), 42.8 (C-24), 33.2 (C-25), 19.7 (C-26), 20.0 (C-27), and 17.6 (C-28). Compound **14** was identified as (3*β*, 5*α*, 6*α*, 14*α*, 22*E*)-3, 14-dihydroxy-5, 6-epoxy-ergosta-8, 22-dien-7-one (Wu et al., 2011).

Among the 13 known compounds, compound **7** and **14** were isolated from *G. lucidum* for the first time. This work further enriched our knowledge about the chemical constituents of *G. lucidum*.

### Anti-proliferative and anti-angiogenic activity

Anti-proliferative activity evaluation against two types of human cancer cells (MDA-MB-231 and HepG2), HUVECs and mouse embryonic fibroblasts (NIH/3T3, used as normal cells) were then carried out. The activities results were shown in Table 2. Compounds **2**, **9–13** showed inhibitory activities against the two tumor cell lines, with IC_50_-values below 100 μM. Especially compounds **9–11** exhibited stronger inhibition against cancer cells compared to the positive control drug kaempferol. Compound **2** exhibited a significant selective inhibition against cancer cells rather than the human umbilical vein endothelial cells (HUVECs). Compounds **3**, **9–13** showed inhibitory activities against HUVECs, with IC_50_-values in the range of 27.9–84.0 μM. Among these bioactive compounds, compounds **9** and **11** showed the strongest activities. Compounds **9–13** showed inhibitory activities against two tumor cell lines as well as HUVECs, which indicated that these four compounds may had multi-target effects on tumor. In additional, all the tested compounds showed no obvious cytotoxicity on the normal cells NIH/3T3, with selective index (SI) greater than 2.

**Table 2:** Anti-proliferative effect of compounds **1**–**14** on different cells.

**No**.	**IC_50_ (μM)**		**EC_50_ (μM)**	
	**HepG2**	**MDA-MB-231**	**HUVEC**	**NIH/3T3**
1	138.3 ± 8.3	176.1 ± 5.1	143.5 ± 6.1	421.6 ± 9.3
2	62.5 ± 1.3	56.3 ± 1.5	142.2 ± 3.6	303.4 ± 11.9
3	129.7 ± 3.4	148.2 ± 5.8	84.0 ± 6.7	364.7 ± 15.7
4	174.6 ± 15.9	148.8 ± 8.7	344.6 ± 13.7	>500
5	196.9 ± 20.1	114.4 ± 9.9	206.3 ± 12.2	>500
6	184.6 ± 9.5	224.2 ± 10.0	384.2 ± 10.9	>500
7	156.4 ± 8.9	168.9 ± 11.1	187.6 ± 15.3	>500
8	286.4 ± 18.9	216.5 ± 14.3	356.7 ± 19.5	>500
9	22.1 ± 0.9	20.3 ± 2.2	27.9 ± 1.7	182.9 ± 10.3
10	50.6 ± 5.3	46.7 ± 5.4	48.7 ± 2.9	194.2 ± 9.6
11	34.8 ± 2.6	44.6 ± 1.9	29.1 ± 3.2	197.4 ± 11.3
12	75.6 ± 4.7	69.7 ± 5.8	72.4 ± 5.6	261.3 ± 12.0
13	44.5 ± 1.3	32.1 ± 3.0	51.6 ± 2.8	293.4 ± 17.2
14	200.9 ± 6.4	189.4 ± 8.1	320.7 ± 18.9	>500
kaempferol*^a^*	45.4 ± 3.4	56.6 ± 3.1	35.5 ± 4.1	356.4 ± 15.9

a *Positive control*.

Previously our research group had found ergosterol peroxide and ergosterol, which were also found in *G. lucidum* in current work (compounds **11** and **12**), could activate Foxo3-mediated cell apoptosis signaling in human hepatocellular carcimoma cells and human breast carcimoma cells (Li et al., 2015, 2016). In present work, we found compounds **11** and **12** inhibited the proliferation of HUVECs, which reminded us that the anti-tumor mechanism of compound **11** and **12** may include multi-targets and multi-approaches except tumor cell apoptosis pathway. Signaling pathways about cell death as well as angiogenesis should be considered. Besides, compound **9** exhibited stronger inhibitory effect against tumor cells than that of ergosterol peroxide (**11**) and ergosterol (**12**). The anti-tumor mechanism of compound **9** *in vitro* and *in vivo* will also be explored in our future work.

### 3D-QSAR

**Figure 5:**
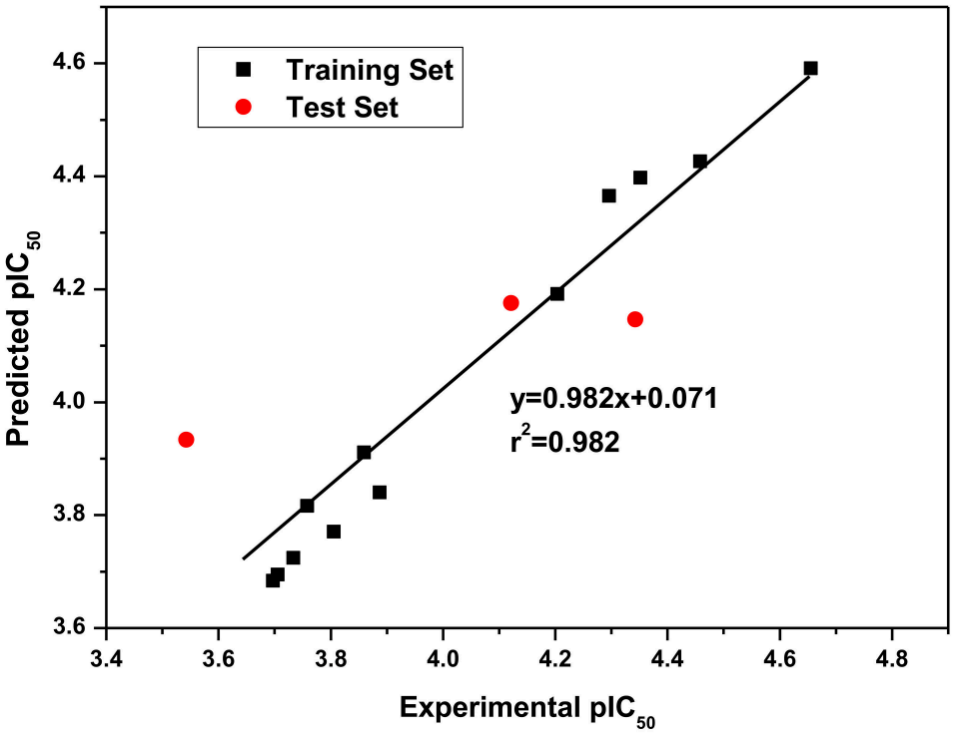
Experimental versus predicted HepG2 inhibitory activities of the training set and the test set. The well agreement between predicted pIC_50_-value and experimental pIC_50_-value for both test sets and training sets indicated that this model was reliable in forecasting activity for the listed compounds.

Since QSAR was a valuable method for novel drug discovery from knowledge in literature and tested bioactive data (Liao et al., 2011), 3D-QSAR was performed for human HepG2 cells. Herein, the selected compounds (**1**–**15**) for model establishment with IC_50_s spanning from 21.2 to 286.4 μM were randomized into training set with the ration of 0.80 and test set of 0.20 by Generate Training and Test Data in DS. *p*IC_50_s converted were between 3.70 and 4.66. The established model presented a good correlation coefficient (*r*^2^) up to 0.982 between observed and predicted activities for training set and 0.861 for test set, which suggested the reliability in external validation. In model generation, 5-fold cross validation of the training set molecules was performed to validating the model, where RMS was at 0.337 and *q*^2^ at 0.002. This suggested the reliability of this model in internal validation. The *p*IC_50_s of assayed and predicted with this model and residual errors were shown (Table 3). Illustratively, a plot was offered (Figure 5), where the correlative relationship between actual and predicted values suggested that this model was reliable for forecasting activities of compounds for HepG2 cells.

**Table 3:** Experimental and predicted inhibitory activities of 15 compounds by 3D-QSAR model against HepG2.

**Compounds**	**Experimental *p*IC50**	**Predicted *p*IC50**	**Residual error**
1	3.85918	3.91081	−0.0516263
2	4.20412	4.19135	0.012774
3	3.88706	3.83999	0.047067
4	3.75796	3.81632	−0.0583582
5	3.70575	3.69473	0.0110238
6	3.73377	3.72433	0.00943665
7	3.80576	3.77072	0.0350384
8^*^	3.54303	3.93341	−0.390383
9	4.65561	4.5912	0.0644135
10	4.29585	4.36558	−0.0697283
11	4.45842	4.42614	0.0322817
12	3.69702	3.68339	0.0136272
13	4.35164	4.39759	−0.0459495
14^*^	4.12148	4.17541	−0.0539262
kaempferol (15)^*^	4.34294	4.14636	0.196585

* *Compounds were selected as the test sets while the rest ones were in the training sets*.

**Figure 6:**
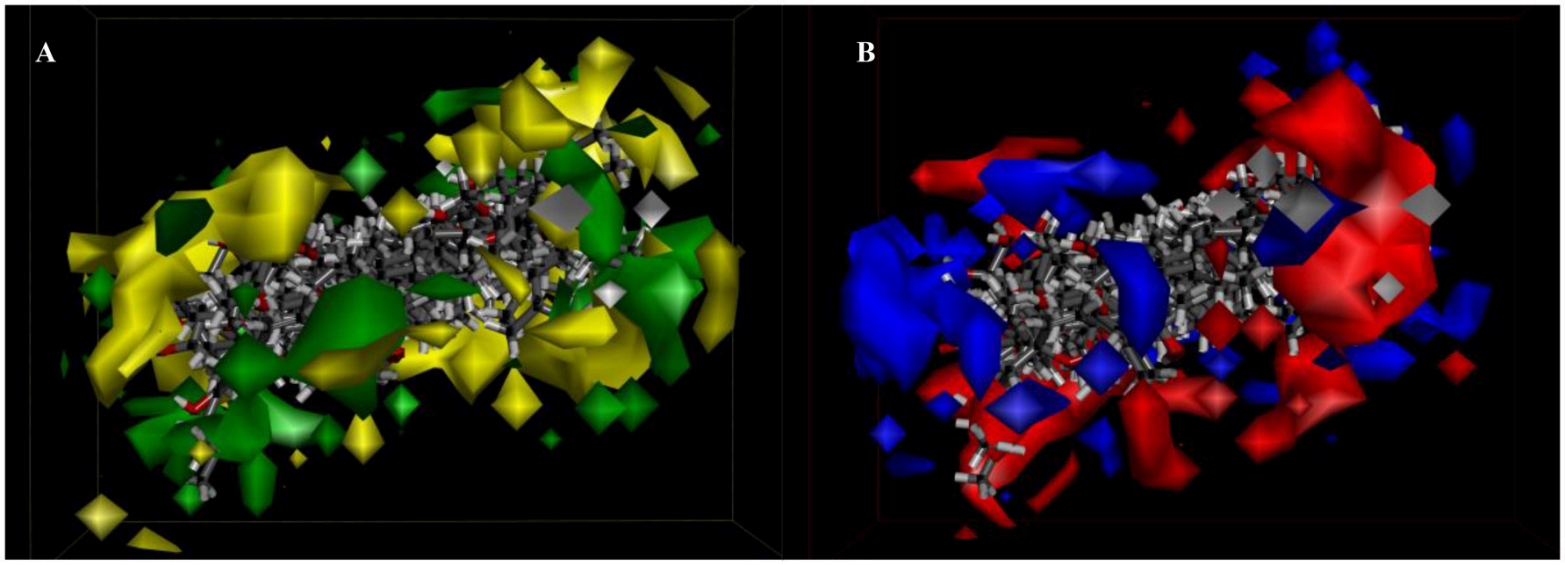
3D-QSAR model. **(A)** 3D-QSAR model coefficients of the listed compounds on van der Waals grids. Green represents positive coefficients; yellow represents negative coefficients. **(B)** 3D-QSAR model coefficients on electrostatic potential grids. Blue represents positive coefficients; red represents negative coefficients.

Besides, *iso*-surfaces of the model on van der Waals (Figure 6A) and electrostatic potential grids (Figure 6B) were provided and aligned by compounds. Accordingly, at C-2, C-3, C-22, and C-28 sites some bulk substituents with slight negative charges might enhance compound 2's activity. Also, at C-20 and C-18 slight large groups with some positive charge could elevate activity for compound 2. But at C-1, C-4, C-11, C-26, and C-27, bulk substitutes might lower activity. Moreover, interchange of C-1, C-2, and C-3 by atoms of negative charge might enhance activity. Evidently, partial hydroxyl negative groups with slight steric hindrance at C-9 and C-14 decline activity to 286.4 μM (IC_50_) for compound **8** (approximately 4.6-folds) in comparison with compound **2** (62.5 μM). However, the introduction of partial hydroxyl negative groups with slight steric hindrance at C-7 and replacement of double hydroxyl groups at C-5 and C-6 with a more slight steric hindrance and more negative epoxy group increase the activity to 22.1 μM for compound **9** (approximately 3-folds) compared with compound **2**. In the words of the most bioactive compound 9, replacement of hydroxyl group at C-7 with a bulky and negative group may increase its activity. Besides, introduction of small positive group at C-28 may elevate its activity. The results will be helpful for designing higher active ergosterol derivatives in our future work.

### Ergosterol content in different parts of *G. lucidum* and products made from *G. lucidum*

Ergosterol, the pro-vitamin D2, is a characteristic secondary metabolite of medicinal and edible fungi, and shows a variety of biological activities. In our previous study, we found that ergosterol purified from *A. rude*, induced tumor cell death and inhibited tumor cell cycle progression, cell migration, and colony growth of MDA-MB-231 cells (Li et al., 2015). Ergosterol prolonged animal survival by upregulating Foxo3 and its downstream molecules Bim, Fas, and Fas L. Ergosterol was also rich in *G. lucidum* and other fungi.

As an important active component in *G. lucidum*, we quantified the contents of ergosterol in different parts of *G. lucidum* and functional foods derived from *G. lucidum* by HPLC. The calibration curves, linear ranges, and recovery of the HPLC method were performed. The linear range of ergosterol was from 20 to 200 μg/mL (*r* = 0. 9994) with an average recovery of 93.32% (RSD was 2.60%, *n* = 9), demonstrating that the HPLC method was precise and accurate enough for quantitative evaluation of ergosterol.

Table 4 showed the content of ergosterol in different parts, including fruiting body, spore and, mycelium of *G. lucidum* and functional foods (spore extract capsule and spore oil capsule) derived from *G. lucidum*. The results suggested the content of ergosterol in different part of raw material of *G. lucidum* was mycelium > fruiting body > spore. Ergosterol in mycelium was about 20 times higher than that in spore. For *G. lucidum* spore products, the ergosterol content was much higher than that of spore raw material. However, the quality homogeneity of *G. lucidum* spore oil capsule products was poor. Considering the significant difference of ergosterol content of *G. lucidum* spore oil capsule, the content variation of *G. lucidum* products may attribute to: (1) Variation of preparation techniques, and (2) Variation of raw materials.

**Table 4:** Contents of ergosterol in different parts and batches of *G. lucidum* preparations.

**Samples**	**Content (μg/g)**	**Samples**	**Content (μg/g)**
FB1	768.0	SE1	1779.4
FB2	697.6	SE2	1633.3
FB3	838.6	SE3	1656.4
MC1	6385.1	SE4	1468.4
MC2	5103.5	SE5	1795.3
MC3	6594.0	SE6	865.4
SP1	361.9	SO1	279.8
SP2	385.8	SO2	2264.5
SP3	246.5	SO3	714.7
SP4	339.5	SO4	1282.9
SP5	369.6	SO5	908.1
SP6	333.0	SO6	2710.0

*FB, fruiting Body; MC, mycelia; SE, Spores extract capsule; SP, Spores powder; SO, Spores oil capsule*.

## Conclusions

Fourteen ergosterol derivatives including a new one were isolated from the lipids enriched fraction of *G. lucidum*. Compounds **9–13** displayed potential inhibitory activity against MDA-MB-231, HepG2, and HUVECs, which indicated that these four compounds had both anti-tumor and anti-angiogenesis activities. Compound **2** had significant selective inhibition against two tumor cell lines, while **3** exhibited selective inhibition against HUVECs. All the compounds showed no obvious cytotoxicity on the normal cells. Their structure–activity relationships for inhibiting HepG2 cells were studied by 3D-QASR. The content of ergosterol in different parts of raw material and preparations of *G. lucidum* were compared, and ergosterol was the highest contented in mycelium. However, the quality homogeneity of *G. lucidum* spore oil capsule products was poor. This study not only enriches the understanding of the diversity of ergosterols in *G. lucidum*, but also provides a basis for further development and utilization of ergosterol derivatives as natural nutraceuticals and functional food ingredients, or as source of new potential antitumor or anti-angiogenesis chemotherapy agent.

## Author contributions

SC was responsible for the concept and design of the study. SC did the isolation and identification of the chemical constituents, evaluated the compounds' activities and wrote the manuscript. TY conducted the QSAR experiment and YZ performed the quantitative analysis. JS, CJ, and YX conducted part of the experiments. All authors participated in the preparation of the manuscript, and have approved the final version.

### Conflict of interest statement

The authors declare that the research was conducted in the absence of any commercial or financial relationships that could be construed as a potential conflict of interest.
