# Supplementary material for: Anti-tumor and Anti-angiogenic Ergosterols from Ganoderma lucidum
Source: Front Chem. 2017 Oct 30;5:85. doi: 10.3389/fchem.2017.00085 (PMC5670154; doi:10.3389/fchem.2017.00085)
Supplement: Supplementary file 1 [file Image1.PDF]

Contents

Fig. S1. ^{1}H NMR spectrum of compound **1** ($CDCl_3$, 600 MHz)

Fig. S2. ^{13}C NMR spectrum of compound **1** ($CDCl_3$, 600 MHz)

Fig. S3. DEPT-135 and ^{13}C NMR spectra of compound **1**

Fig. S4. ^{1}H-^{1}H COSY spectrum of compound **1**

Fig. S5. HSQC spectrum of compound **1**

Fig. S6. HMBC spectrum of compound **1**

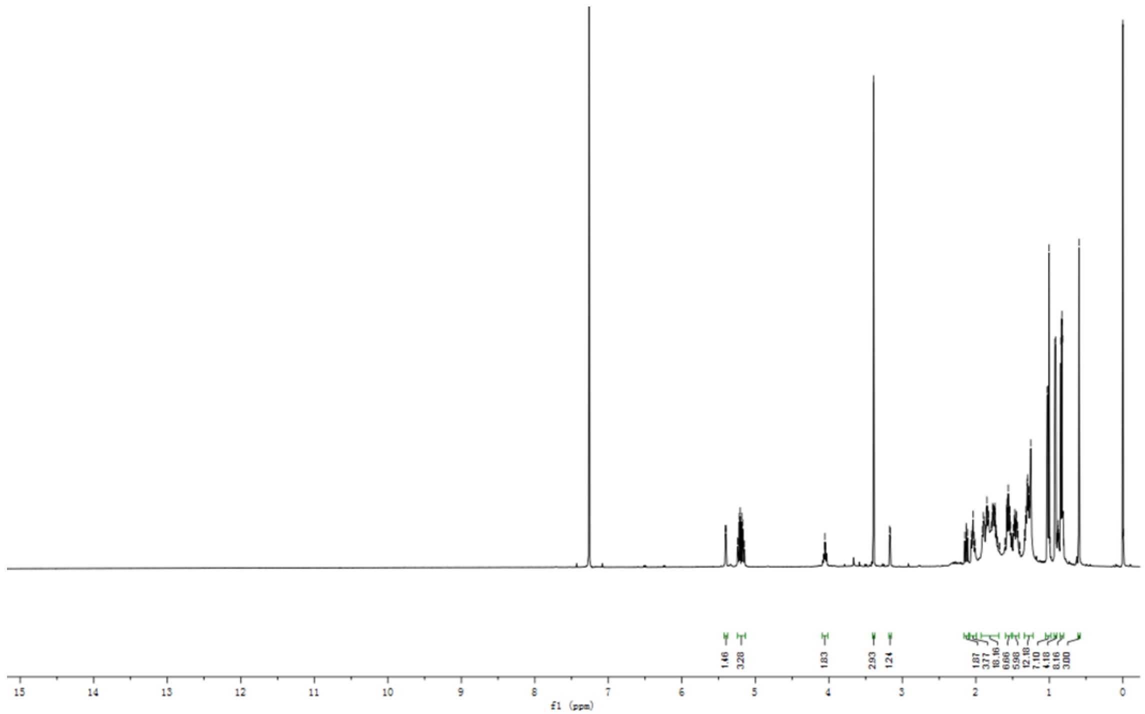

Fig. S1. ^{1}H NMR spectrum of compound **1** ($CDCl_3$, 600 MHz)

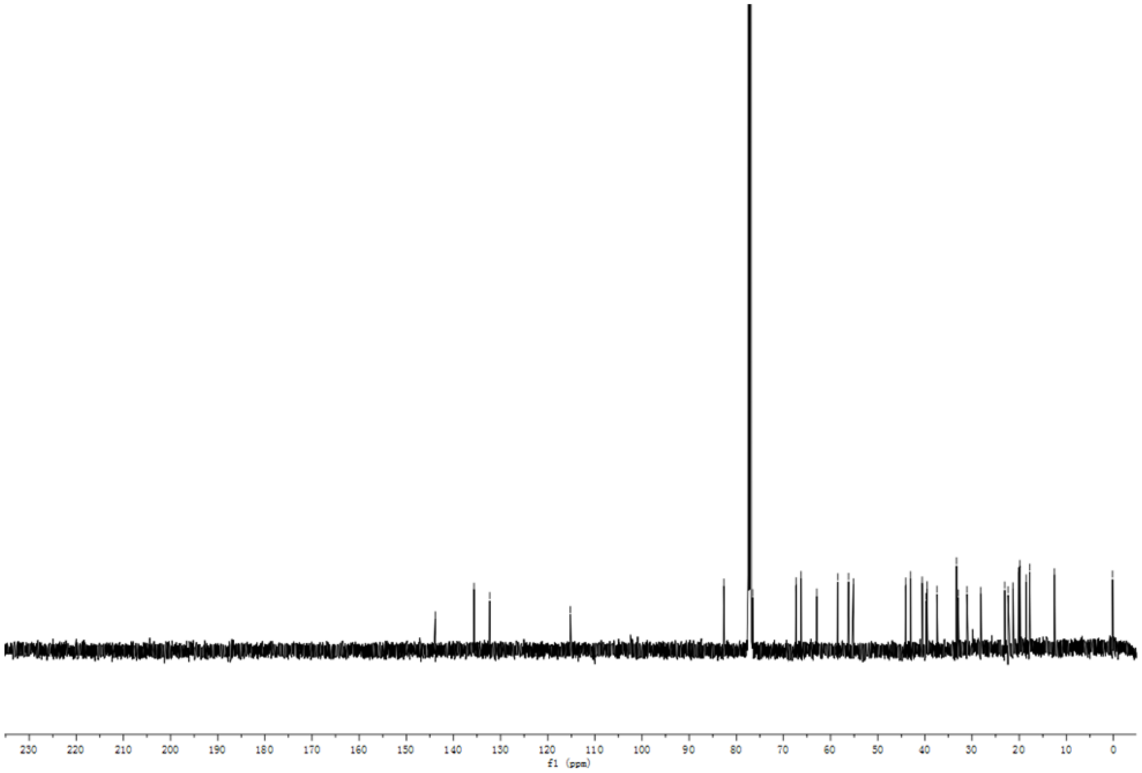

Fig. S2. ^{13}C NMR spectrum of compound **1** ($CDCl_3$, 600 MHz)

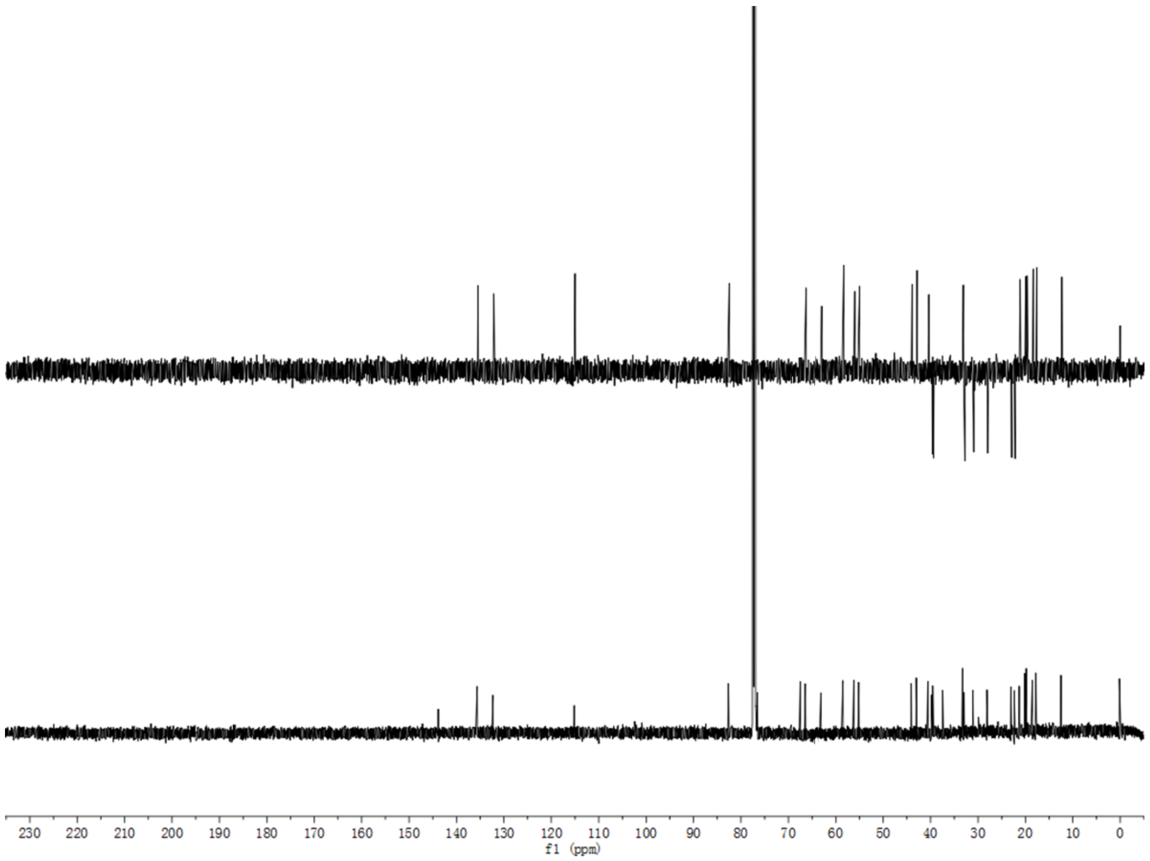

Fig. S3. DEPT-135 and ^{13}C NMR spectra of compound **1**

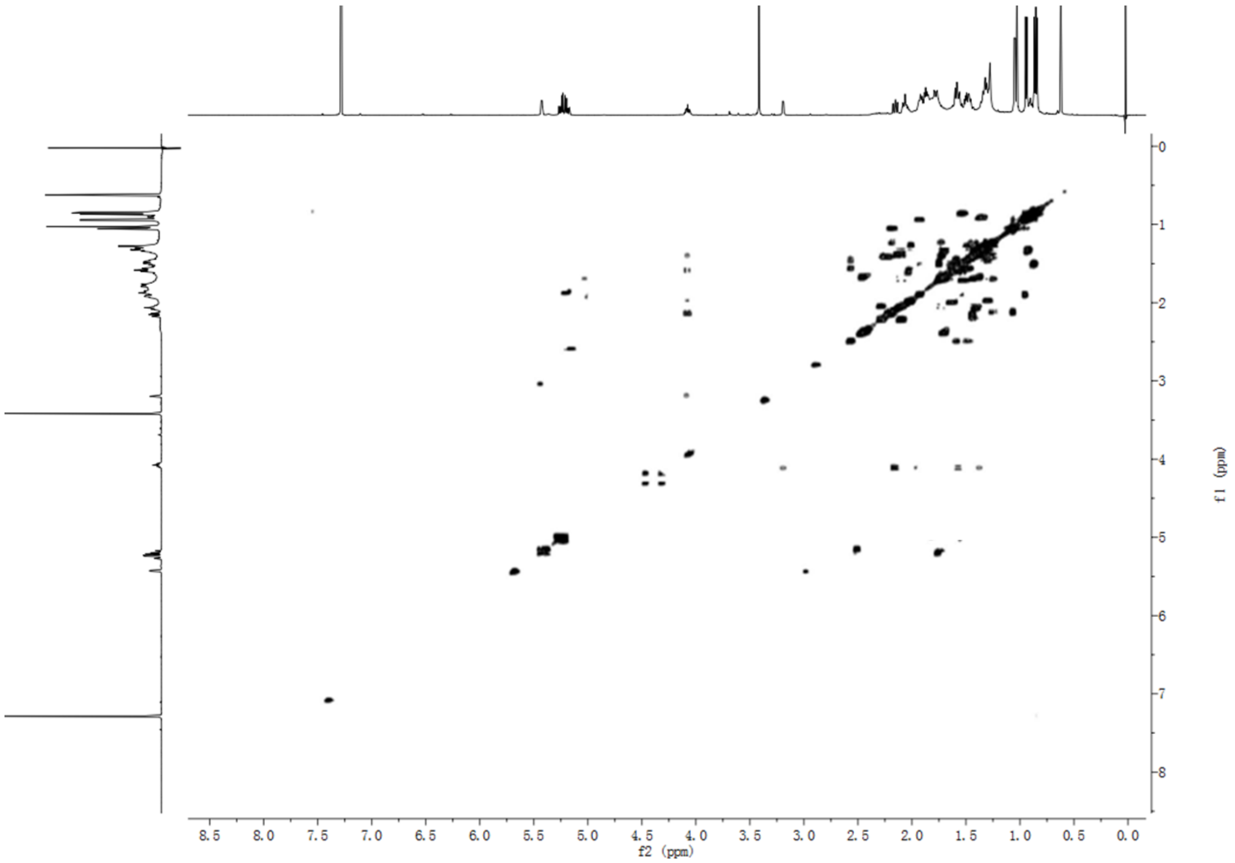

Fig. S4. ^{1}H-^{1}H COSY spectrum of compound **1**

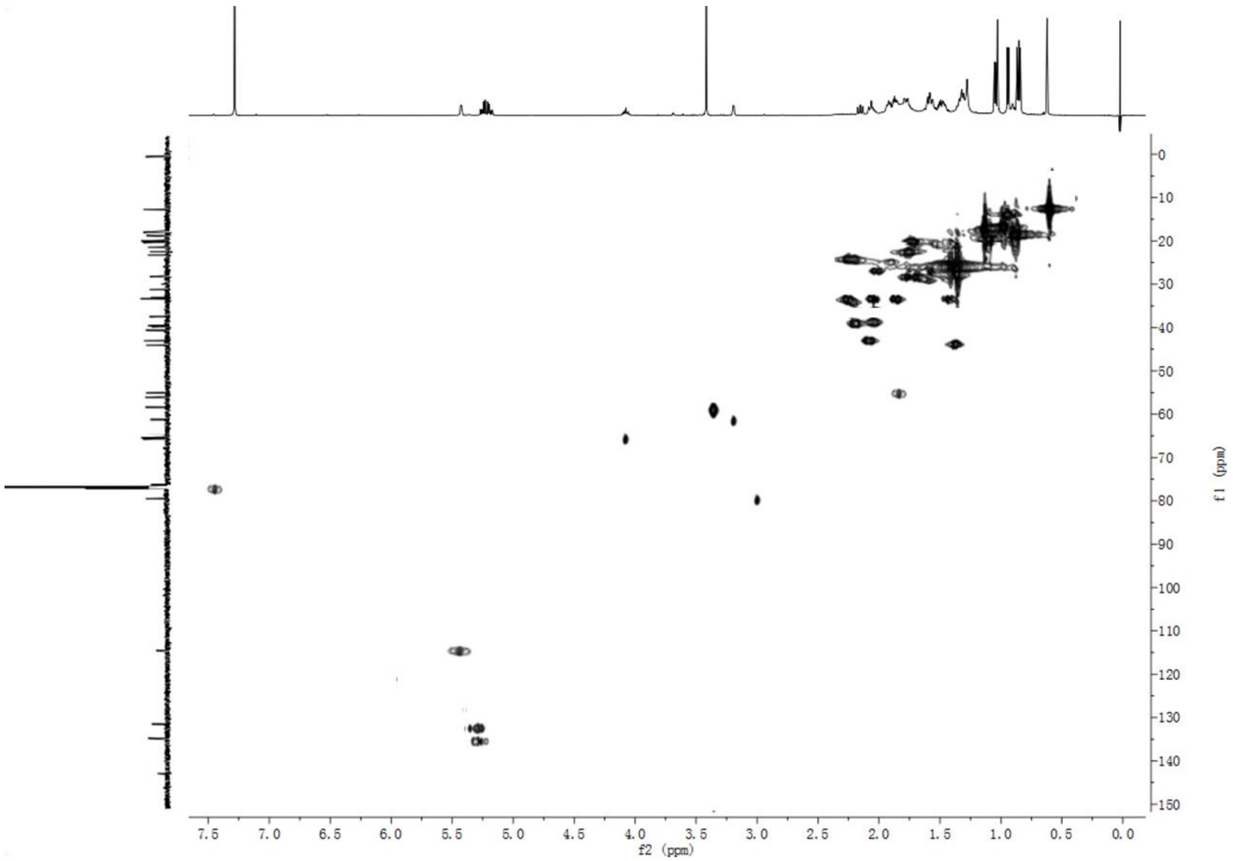

Fig. S5. HSQC spectrum of compound **1**

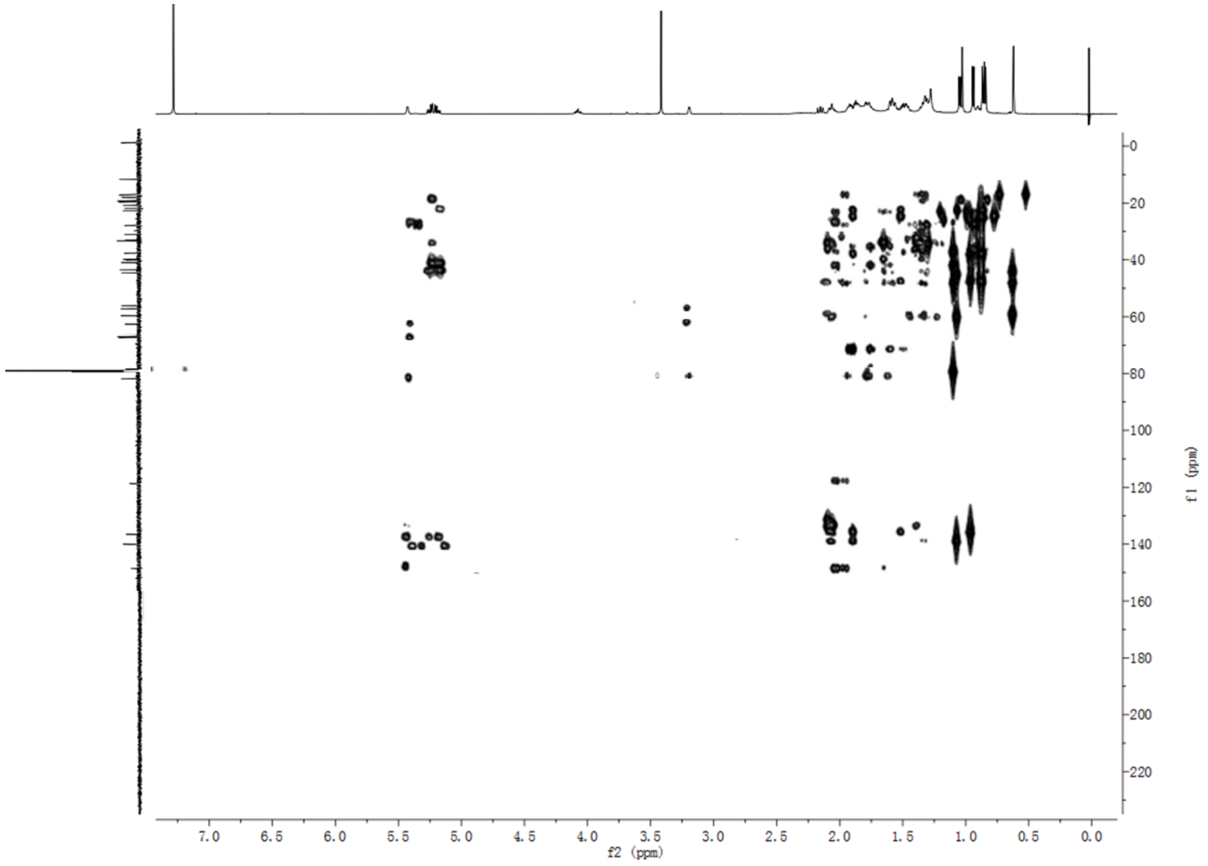

Fig. S6. HMBC spectrum of compound **1**
